# MV CBCT-Based Synthetic CT Generation Using a Deep Learning Method for Rectal Cancer Adaptive Radiotherapy

**DOI:** 10.3389/fonc.2021.655325

**Published:** 2021-05-31

**Authors:** Jun Zhao, Zhi Chen, Jiazhou Wang, Fan Xia, Jiayuan Peng, Yiwen Hu, Weigang Hu, Zhen Zhang

**Affiliations:** ^1^ Department of Radiation Oncology, Fudan University Shanghai Cancer Center, Shanghai, China; ^2^ Department of Oncology, Shanghai Medical College, Fudan University, Shanghai, China; ^3^ Shanghai Key Laboratory of Radiation Oncology, Shanghai, China; ^4^ Department of Medical Physics, Shanghai Proton and Heavy Ion Center, Shanghai, China

**Keywords:** MV CBCT, CycleGAN, synthetic CT, adaptive radiotherapy, rectal cancer

## Abstract

Due to image quality limitations, online Megavoltage cone beam CT (MV CBCT), which represents real online patient anatomy, cannot be used to perform adaptive radiotherapy (ART). In this study, we used a deep learning method, the cycle-consistent adversarial network (CycleGAN), to improve the MV CBCT image quality and Hounsfield-unit (HU) accuracy for rectal cancer patients to make the generated synthetic CT (sCT) eligible for ART. Forty rectal cancer patients treated with the intensity modulated radiotherapy (IMRT) were involved in this study. The CT and MV CBCT images of 30 patients were used for model training, and the images of the remaining 10 patients were used for evaluation. Image quality, autosegmentation capability and dose calculation capability using the autoplanning technique of the generated sCT were evaluated. The mean absolute error (MAE) was reduced from 135.84 ± 41.59 HU for the CT and CBCT comparison to 52.99 ± 12.09 HU for the CT and sCT comparison. The structural similarity (SSIM) index for the CT and sCT comparison was 0.81 ± 0.03, which is a great improvement over the 0.44 ± 0.07 for the CT and CBCT comparison. The autosegmentation model performance on sCT for femoral heads was accurate and required almost no manual modification. For the CTV and bladder, although modification was needed for autocontouring, the Dice similarity coefficient (DSC) indices were high, at 0.93 and 0.94 for the CTV and bladder, respectively. For dose evaluation, the sCT-based plan has a much smaller dose deviation from the CT-based plan than that of the CBCT-based plan. The proposed method solved a key problem for rectal cancer ART realization based on MV CBCT. The generated sCT enables ART based on the actual patient anatomy at the treatment position.

## Introduction

Neoadjuvant chemoradiotherapy, which can improve the local control rates, is a standard of care for locally advanced rectal cancer (1, 2). Using the high conformal radiation technique, intensity modulated radiotherapy (IMRT) or volumetric modulated radiotherapy (VMAT) can provide target high dose distribution while better sparing surrounding normal tissues than 3D conformal radiotherapy (3, 4). Most current treatment strategies use one treatment plan based on the pretreatment CT throughout the whole treatment period with or without image guide radiotherapy (IGRT). However, due to the differences in bladder and rectal filling status, the shape and position of the rectum and mesorectum may change during radiation therapy (5, 6). This could cause the target volume to be missed or a high dose to be delivered to the surrounding normal tissues during radiation therapy, resulting in loss of local control or serious side effects. Zumre et al. conducted a study on rectal cancer patients treated with neoadjuvant radiotherapy to evaluate mesorectum movement and its effect on dose distribution. The study revealed 20 mm of mesorectum movement in the lateral and anterior-posterior direction and 10 mm of movement in the superior-inferior direction during radiotherapy, which caused a median of ~2% change in dosimetric parameters (7). A larger planning target volume (PTV) margin can ensure that no target is missed but will deliver a high dose to normal tissue. A smaller PTV margin can better protect normal tissues but may result in a prescription dose that misses the target volume. Adaptive radiotherapy (ART), which takes into account the anatomy changes of the patient during treatment, is the best way to solve this problem. One study introduced plan selection strategies to account for the anatomy changes during rectal radiotherapy (8). They created three treatment plans according to three different PTV margins regarding three different filling states of the bladder (full, empty and intermediate state). Then, the best plan for treatment was chosen according to online cone beam CT (CBCT). Another study compared an online adaptive radiotherapy strategy for planning the selection with respect to the dose to the organ at risk for rectal cancer (9), and they found that the adaptive treatment maintained target coverage and reduced the doses to the organs at risk (OARs). Both of these strategies are superior to using one plan throughout the whole course, but they all have limitations in that the calculation of the dose distribution was based on the planning CT rather than on the online patient anatomy; rather, they simply take into account the delineation on the online CBCT. To fulfill the ART process, we should directly use the images with actual online anatomy for dose calculation.

Online CBCT represents the actual patient anatomy at the treatment positions that are mostly used for image-guided radiotherapy. Due to its image quality limitation, CBCT cannot be used for dose calculation directly. Several traditional methods are used to improve CBCT image quality to make it suitable for dose calculation, such as using the deformed CT as previously described for scatter correction of the CBCT projection (10). Some others use an anti-scatter grid or different scatter kernel algorithms for deconvolving scatter from projections (11, 12). In recent years, deep learning methods have been widely used for medical image modality transformation to generate synthetic CT (sCT) images (13–16). Cycle-consistent adversarial network (CycleGAN) is one of the most commonly used methods for image transformation, as it does not require paired information of the training data (17). In a real clinical situation, it is almost impossible to obtain paired images. Thus, CycleGAN is a perfect tool for the CBCT to sCT transformation. Several groups have successfully used this method for MR to CT transformation and CBCT to CT transformation (18–24). It is proved that the CycleGAN performed better than other supervised learning methods, such as deep convolutional generative networks (DCGAN), progressive growing of GANs (PGGAN) and U-Net (18, 21). In these studies, most of them chose head and neck tumor sites for research, which has a relatively stable anatomy. Fewer studies have evaluated the usage of CycleGAN in the abdominal and pelvic regions, where the organs usually have larger positional deviation and shape changes. Moreover, no studies have evaluated the image transformation from Megavoltage (MV) CBCT to CT, and no studies have evaluated the use of CycleGAN for rectum tumor sites.

In this study, we aim to use the CycleGAN model to transform MV CBCT images into sCT images of rectal cancer patients and to evaluate whether the synthetic image is sufficient for ART through image quality evaluation, autosegmentation capacity and dose calculation capacity evaluation.

## Materials and Methods

### Image Acquisition and Processing

In this study, a newly designed CT-linac uRT-linac 506c was used for CT and CBCT data acquisition. The CT-linac is a new product of United Imaging Healthcare (UIH) Co., Ltd, which integrated a diagnostic-quality 16–slice helical CT and a C-arm linac together. The helical CT can be used for simulation or IGRT. The linac also has an electronic portal imaging detector (EPID) system for 2D portal image and 3D MV CBCT acquisition.

There were 40 rectal cancer patients involved in this study. The patients’ age range from 38 to 70 with a median age of 58. For each patient, the IMRT technique incorporated with image guidance was used for treatment. Image guidance was performed every day in the first 3 fractions and then once a week. In the image guide process, FBCT was acquired for position correction, and then the MV CBCT was acquired for position verification. Thus, we were able to acquire online CT and CBCT image pairs with almost the same position and the same anatomy. One hundred image pairs of 30 patients were used for model training, and 10 image pairs of the remaining 10 patients were used for evaluation.

The CBCT and CT images were preprocessed before the model training, which can eliminate the impact of the non-anatomical structure. All the images were resampled to the same resolution of 0.8789 mm by 0.8789 mm and a slice thickness of 3 mm, and all of them were cropped to the size of 512 * 512. Each patient’s CBCT and CT images were aligned with each other, and the image slices that existed in both CBCT and CT were selected as training and validation data. Binary masks were generated using an Otsu autothresholding method to separate the inside and outside body regions, and for the outside body area, each voxel value was assigned as -1000. To speed up the training convergence, we scaled the CBCT and CT image values to the range of (-1, 1) according to the formula $I_{s}=2*\frac{\left(I_{orig}+1000\right)}{4095}-1,$ where *I_orig_* indicates the original CBCT and CT images, whose value range is (-1000, 3095).

### CycleGAN-Based CBCT to sCT Generation

**Figure 1:**
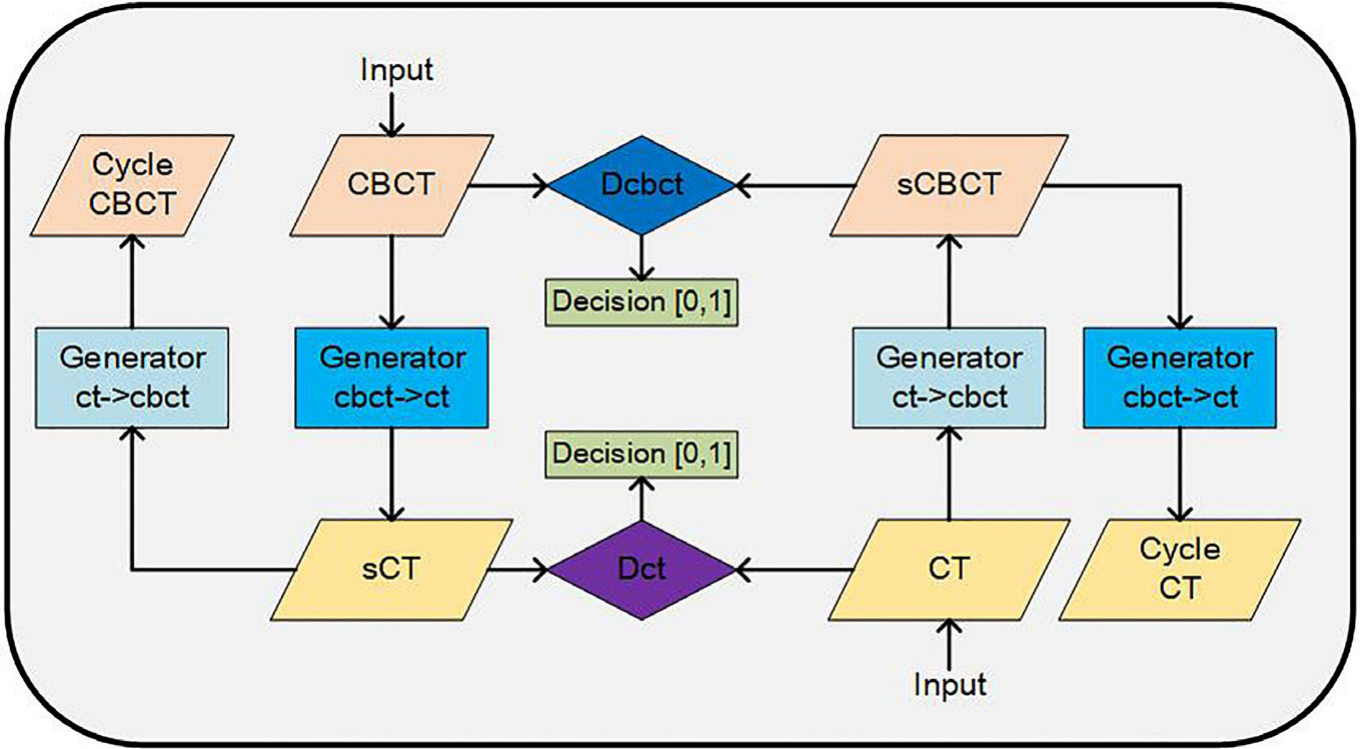
Architecture of the CycleGAN network for image synthesis.

The architecture of CycleGAN is shown in **Figure 1**. The main structure of the CycleGAN contains 4 parts: 2 generators, *G_cbct→ct_*, which can convert a CBCT image into a synthesized CT, and *G_ct→cbct_*, which can synthesize a CBCT image from a CT; 2 discriminators, *D_cbct_*, which distinguishes synthesized CBCT images from real CBCT images, and *D_ct_*, which identifies the synthesized CT images from real CT images. The architectures of the generator and discriminator are both borrowed from Kida’s research (22) with a few changes. In the generator, we used U-net structure instead of the encoder-decoder because the U-net can better maintain the anatomy of the CBCT images according to our experience. By using the encoder-decoder structure, some air pockets near the femoral heads and caudal vertebra in the sCT images would be generated that did not exist in the CBCT images.

**Figure 2:**
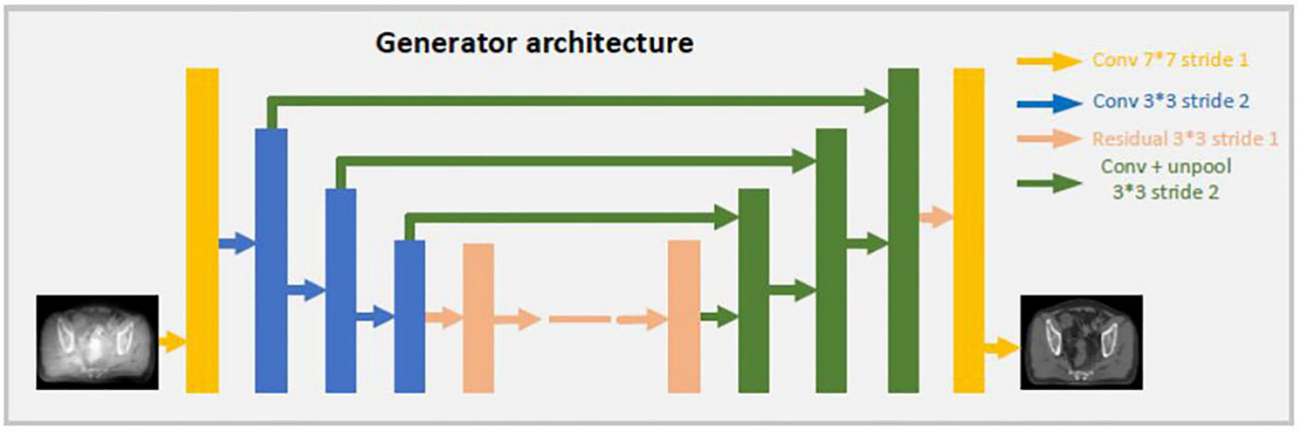
Architecture of the generator.

Both generators share the same U-net network, the details are showed as **Figure 2**. CBCT (or CT) images are the inputs of the model, and the synthetic CT (or synthetic CBCT) images are outputs. The U-net network contains one convolution layer with a 7 * 7 kernel with stride 1; three down convolution layers with a 3 * 3 kernel with stride 2 and channels 32, 64 and 128; 9 residual blocks with a 3 * 3 kernel with stride 1; three up-sampling layers each consisting of an unpooling with stride 2; a residual block with a 3 * 3 kernel with stride 1 and a convolution layer with a 7 * 7 kernel with stride 1.

**Figure 3:**
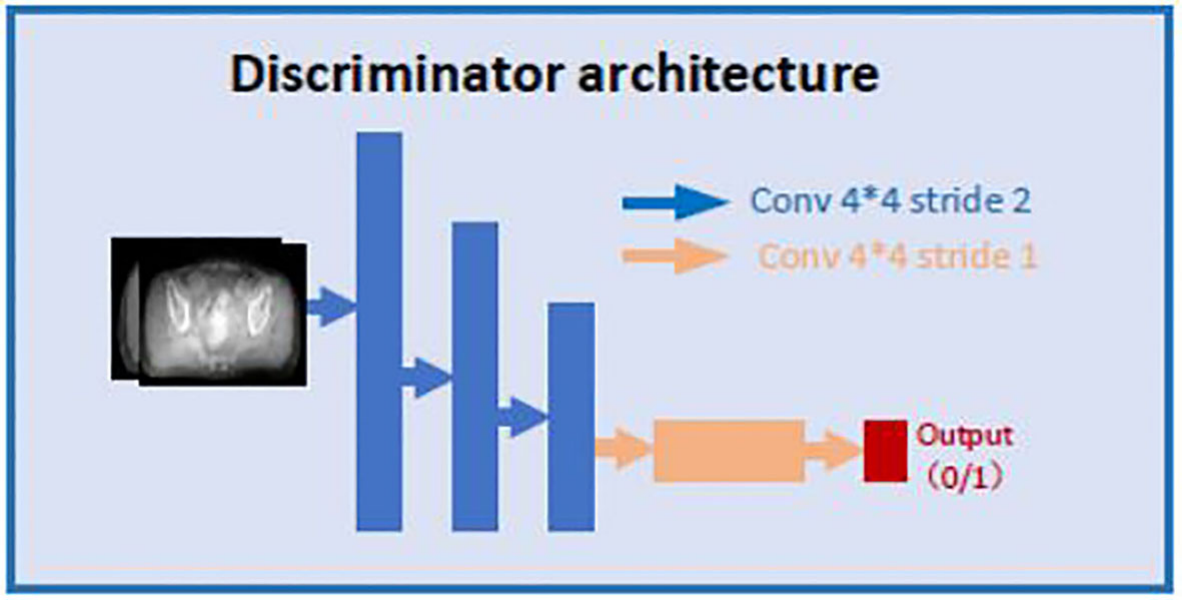
Architecture of the discriminator.

Both discriminators use the same architecture as shown in **Figure 3**. CBCT (or CT) images are set as input data. The discriminator includes 3 down convolution layers with a 4 * 4 kernel with stride 2 and channels 32, 64 and 128; a convolution layer with a 4 * 4 kernel with stride 1 and channel 256; and a convolution layer with a 4 * 4 kernel with stride 1 and channel 1. The last layer will be compared with a same shape of array filled with 0 or 1 to determine whether the input image is fake or real.

The loss functions were also adapted from Kida’s research (22). There are two loss functions, *Loss_D_* and *Loss_G_*, for the discriminator and generator models, respectively. *Loss_D_* istrying to distinguish the real CT and CBCT images from synthesized ones, while *Loss_G_* is trying to minimizing the error between the synthesized image and the real image. *Loss_G_* consists of several items as follows:

$$Loss_{G}=\;\lambda_{cycle}Loss_{cycle}+\lambda_{idem}Loss_{idem}+\lambda_{adv}Loss_{adv}+\lambda_{grad}Loss_{grad}$$

*Loss_cycle_* ensures that the synthesized cycle images is closed to the original images. *Loss_idem_* makes sure the generator G and *G* ^2^ are idempotent and helps to increase the stability during training. *Loss_adv_* encourages the generator to generate a synthesized image that is as close to a real image as possible. *Loss_grad_* encourages structural preservation before and after conversion by trying to keep the edges in the image.

The Adam optimizer was used to train the model with a batch size of 1. The hyperparameters *λ_cycle_*, *λ_idem_*, *λ_adv_* and *λ_grad_* were set to 20, 1, 1 and 1, respectively. For the training from scratch, the learning rate was set as 10^-4^. All the implementations used Python 3.6 with a chainer. All experiments were performed on a Linux workstation with one NVIDIA GEFORCE RTX 2080TI. The training required approximately 6 days for 100 epochs, and the prediction (including preprocessing) required approximately 10 s for one set of CBCT images.

One should pay attention to the overfitting problem caused by small training data size in the model training. Usually, the dropout method and data augmentation can be used to avoid overfitting. In this study, although we have about 9000 images in each training data set, we added noise to the input data during training to avoid overfitting.

### Synthetic CT Image Quality Evaluation

In this study, CBCT and CT images of 10 patients were used for the model performance evaluation. For the generated synthetic image quality evaluation, we used mean absolute error (MAE), peak signal-to-noise ratio (PSNR), normalized cross-correlation (NCC), and structural similarity (SSIM) as evaluation indices (18).

(1) $$\text{MAE}(I_{1},I_{2})=\frac{1}{n_{i}n_{j}n_{k}}\sum_{x,y,z}^{n_{i}n_{j}n_{k}}\left|I_{1}(x,y,z)-I_{2}(x,y,z)\right|$$

(2) $$\text{PSNR}(I_{1},I_{2})=10\times \;\log_{10}\left(\frac{MAX^{2}}{\sum_{x,y,z}^{n_{i}n_{j}n_{k}}{\left|I_{1}(x,y,z)-I_{2}(x,y,z)\right|}^{2}/n_{i}n_{j}n_{k}}\right)$$

(3) $$\text{NCC}(I_{1},I_{2})=\frac{1}{n_{i}n_{j}n_{k}}\sum_{x,y,z}^{n_{i}n_{j}n_{k}}\frac{\left(I_{1}\left(x,y,z\right)-\mu_{I_{1}}\right)\left(I_{2}\left(x,y,z\right)-\mu_{I_{2}}\right)}{\sigma_{I_{1}}\sigma_{I_{2}}}$$

(4) $$\text{SS}I\text{M}(I_{1},I_{2})=\frac{\left(2\mu_{I_{1}}\mu_{I_{2}}+c_{1}\right)\left(2\sigma_{I_{1},I_{2}}+c_{2}\right)}{\left(\mu_{I_{1}}^{2}+\mu_{I_{2}}^{2}+c_{1}\right)\left(\sigma_{I_{1}}^{2}+\sigma_{I_{2}}^{2}+c_{2}\right)}$$

*I*_1_ and *I*_2_ represent two different images. *I* (*x, y, z*) means the HU value of pixels (*x, y, z*) in image I. *n_i_n_j_n_k_* is the total number of pixels in image I. *MAX* is the maximum HU value in the selected image. µ and σ represent the mean and the standard deviation of the HU value in an image. Online fan beam CT was the ground truth image for comparison. sCT and CBCT images were compared with fan beam CT.

### Autosegmentation and Dosimetric Evaluation of Synthetic CT

Autosegmentation capability, which can improve the segmentation efficiency, is crucial for the ART process. In our clinical situation, the autosegmentation model trained using CT images in UIH TPS was regularly for rectal cancer patients’ target and organ delineation. To evaluate the performance of the autosegmentation model on sCT can indirectly evaluate the similarity between CT and sCT and check whether this autosegmentation model is suitable for sCT to improve the efficiency of ART process. So the segmentation model was used to delineate target and organ at risk on sCT. Then, the contours were reviewed and modified by an experienced physician on the sCT. The autocontours and manually modified contours were compared using dice similarity coefficients (DSC) to evaluate the autosegmentation accuracy on sCT.

In order to evaluate the performance difference of the autosegmentation model on sCT and CT. The segmentation model was used to delineate target and organ at risk on CT. Then these contours were transformed to the corresponding sCT. DSC index was used to evaluate the similarity between the autocontours from sCT and CT. The following formula was used to calculate DSC, in which V_1_ and V_2_ represents the volume of the two contours for comparison respectively.

(5) $$\text{DSC}=\;\frac{2\left(V_{1}\;\cap V_{2}\right)}{V_{1}V_{2}}$$

Dose calculation capability is also very important of sCT for the ART accuracy. So the autoplanning function in UIH TPS was used to generate IMRT treatment plan on sCT to check whether clinical acceptable plans can be generated. The manually modified contours on sCT were used for planning. Then the plan and contours were transferred to the corresponding CT. The dose volume histogram was used to evaluate the dose distribution difference between sCT and CT based plans. *V*95%, *V*100% (volume of the target receiving at least 95% and 100% of the prescribed dose), D99, D5, D95 (doses to 99%, 5% and 95% of the volume) and *Dmean* (mean dose of the volume) were investigated for PTV (25). For OARs, volumes receiving different dose levels were evaluated. The dose volume statistics of V30, V40 and V50 for bladder and V30 and V40 for femoral heads were investigated (Vx means the percentage of volume receiving xGy dose). As comparison, the plan and structure were also transferred to CBCT and these dosimetric differences were also compared between CBCT-based and CT-based plans.

## Results

**Figure 4:**
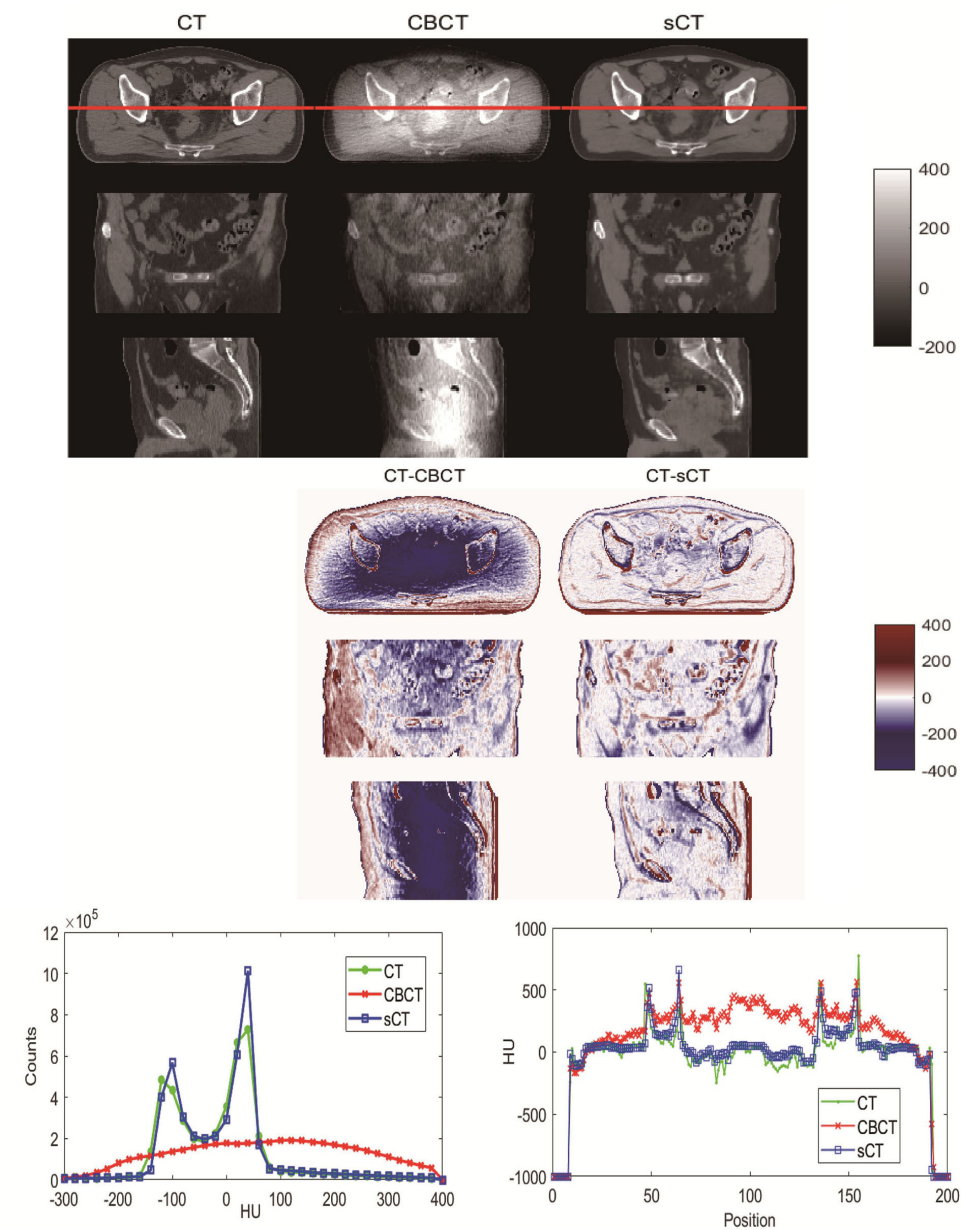
Visual comparison of CT, CBCT and sCT images of one patient. The HU difference between two image sets, HU histogram comparisons and line profile comparisons for CT, CBCT and sCT images.

Visual comparisons of CBCT and sCT with CT images are shown in **Figure 4**. The HU difference between two image sets, the HU histogram comparisons and one line profile comparisons for CT, CBCT and sCT images are also shown in **Figure 4**. We can see that the sCT image quality was greatly improved over that of CBCT images and was very close to the quality of the CT images. The sCT images reduced the scatter artifacts while retaining the anatomical accuracy and sharpening the boundaries of the soft tissue structures. The HU histogram and the line profile of the three different image modalities shown in **Figure 4** reveal great improvement of the HU value from CBCT to sCT. Additionally, the HU histogram of sCT is in good agreement with that of CT.

For image quality analysis, CBCT and sCT images were compared with CT images using MAE, PSNR, NCC and SSIM. The results are listed **Table 1**. From the results, we can see that the image quality of sCT images generated by the CycleGAN model was noticeably improved, and the images were more similar to real CT images.

**Table 1:** Numerical comparisons of CBCT and sCT with CT images.

	CBCT *vs* CT	sCT *vs* CT
MAE (HU)	135.84 ± 41.59	52.99 ± 12.09
PSNR(dB)	21.76 ± 1.95	26.99 ± 1.48
NCC	0.96 ± 0.01	0.98 ± 0.01
SSIM	0.44 ± 0.07	0.81 ± 0.03

MAE, mean absolute error; PSNR, peak signal-to-noise ratio; NCC, normalized cross-correlation; SSIM, structural similarity; sCT, synthetic CT.

**Figure 5:**
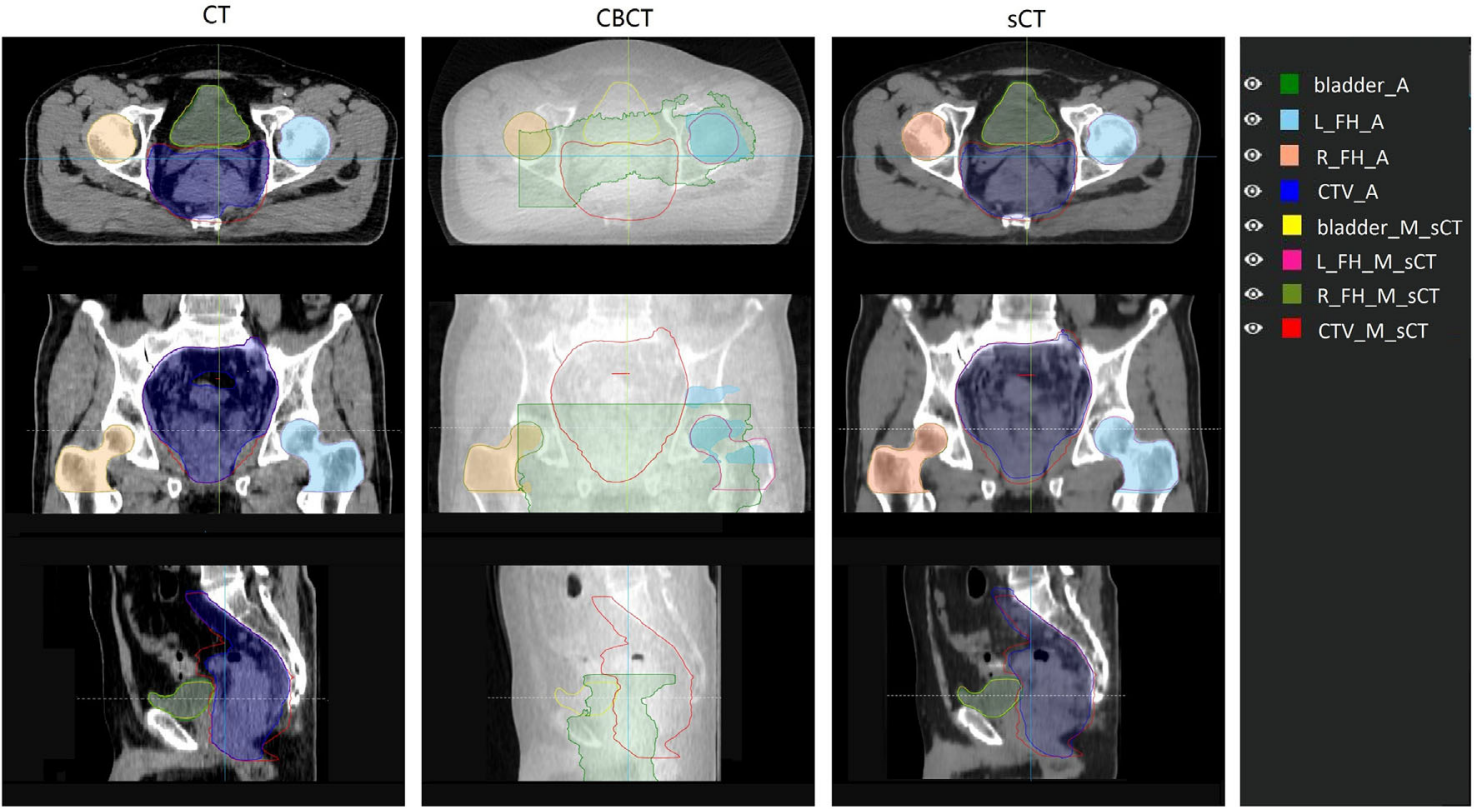
Comparison of auto contour on different image set with the manual contour delineated on sCT. The manual contour was delineated on sCT and then copied to CT and CBCT. Contour name with “_A” suffix represents auto contour. Contour name with “_M_sCT” suffix represents contour manually delineated on sCT. L_FH and R_FH means left femoral head and right femoral head.

For the autosegmentation capability evaluation of sCT images, the DSC index was used to compare the similarity between contours. The results are shown in **Table 2**. From the comparison between autocontours and manual-modified contours on sCT, we can see that the auto-segmentation model performance for femoral heads was very accurate and needed almost no manual modification for the auto-contours. For the CTV and bladder, we can see that although modification is needed for the auto-contour, the DSC indices were high, at 0.93 and 0.94 for the CTV and bladder. These findings indicate that the autosegmentation is accurate enough for clinical use to improve segment efficiency while retaining accuracy on sCT. From the comparison between autocontours on sCT and CT, it can be seen that they have high DSC index. On the contrary, the autosegmentation model is almost not capable of segmenting the CTV and bladder on CBCT. Even the autosegmentation of femoral head on CBCT has large error. An example of autosegmentation on CT, CBCT and sCT and their difference with manual-modified contour delineated on sCT is shown in **Figure 5**. It visually revealed the capability of autosegmentation on sCT and CT. And we can see that the CTV cannot be delineated by autosegmentation and the bladder is delineated totally wrong on CBCT. The delineation of the left femoral head on CBCT is also with big error.

**Table 2:** Comparison of the similarity between contours.

	CTV	bladder	L_FH	R_FH
DSC (auto vs manual)	0.93 ± 0.04	0.94 ± 0.08	0.99 ± 0.01	0.99 ± 0.02
DSC (sCT vs CT)	0.95 ± 0.03	0.89 ± 0.03	0.93 ± 0.04	0.95 ± 0.02

DSC (auto vs manual), DSC index between autocontours and manual-modified contours on sCT; DSC (sCT vs CT), DSC index between autocontous on sCT and CT; L_FH, left femoral head; R_FH, right femoral head.

**Figure 6:**
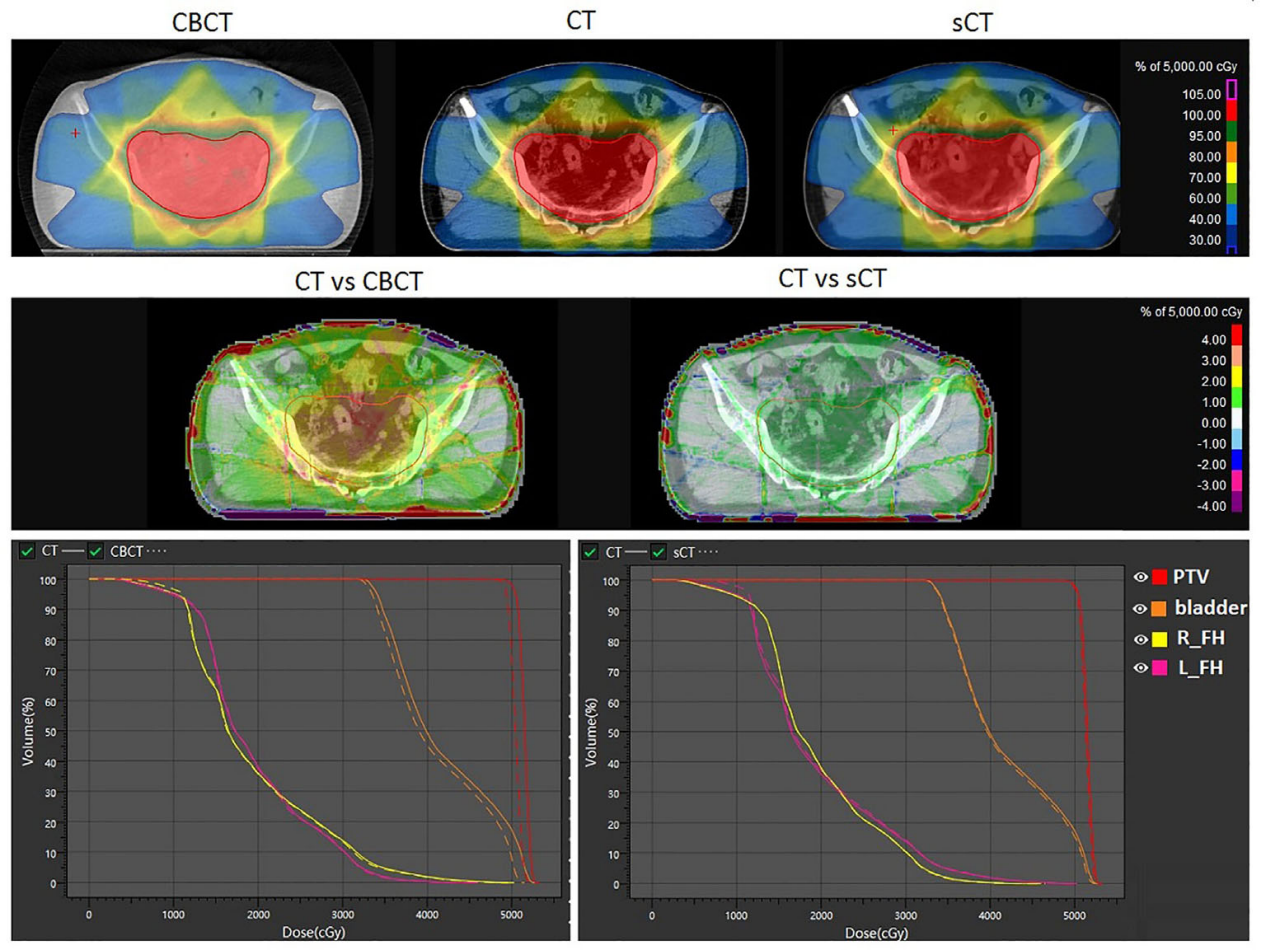
Dosimetric comparison of the same plan calculated on CBCT, sCT and CT. The first row shows the dose distribution on CBCT, CT and sCT. The second row shows the dose distribution differences. The third row shows the DVH differences. L_FH and R_FH means left femoral head and right femoral head.

According to our experiment, the autoplanning function of UIH TPS is capable of generating clinical acceptable plans for rectal cancer radiotherapy on sCT. The results of the dose calculation accuracy evaluation of sCT are shown in **Figure 6**. The first row of **Figure 6** shows the dose distribution of the same plan on CBCT, CT and sCT for individual patient. The second and third row of **Figure 6** shows the difference of CBCT- and sCT- based plan compared with CT-based plan in terms of dose distribution and dose volume histogram (DVH) respectively. From **Figure 6**, we can see that the DVH values of CT- and sCT-based plans have small differences, and the DVH lines almost overlap, while the DVH comparison reveals a larger dose difference of the PTV and bladder between CT- and CBCT-based plans. The second row shows the dose distribution differences on one axial slice. The dose difference between CT- and CBCT-based plans can be up to 4% in PTV, while the difference between CT- and sCT-based plans was reduced to within 1% in PTV. For both of these comparisons, we can see larger dose differences at the boundary of the body. This is caused by the image boundary difference between CT and CBCT, which can also be seen in **Figure 4** with a large HU difference at the body boundary. The respiratory motion during the long scanning time of CBCT resulted in anterior and lateral boundary differences between CBCT and CT images. The posterior boundary difference may caused by the outer boundary delineation inaccuracy due to couch scattering on CBCT during preprocessing.

**Figure 7:**
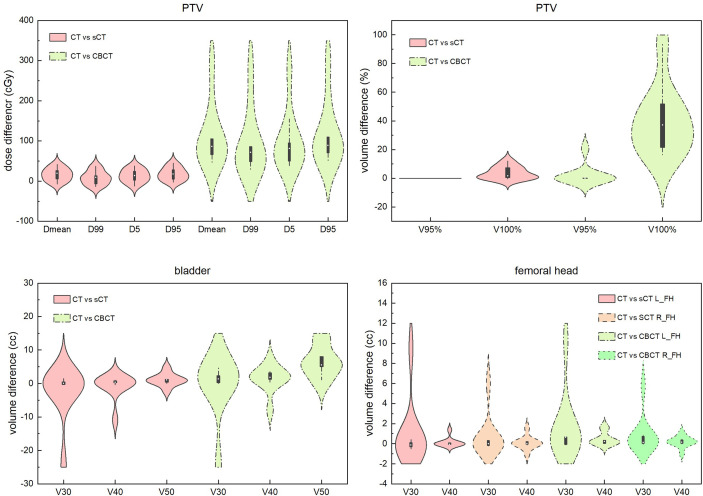
Dosimetric difference comparisons of sCT- and CBCT-based plans with CT-based plans for PTV, bladder and the femoral head. Dmean represents the mean dose to the volume. Dx represents the dose to x% of the volume. Vx% represents the volume receiving at least x% of the prescribed dose. Vx represents the percentage of volume receiving xGy dose. L_FH and R_FH means left femoral head and right femoral head.

Except for the direct view of DVH and dose distribution comparisons, we systematically compared some critical dose statistical differences in sCT- and CBCT-based plans with CT-based plans for PTV, bladder and the femoral head. The results are shown in **Figure 7**. We can see that for PTV dose statistics, the differences in *Dmean*, D99, D5 and D95 between the CT- and sCT-based plans are mostly less than 50 cGy, which are smaller than the differences between CT- and CBCT-based plans. The dose difference between CT- and CBCT-based plans could be high as 350 cGy. For the comparison of PTV volume receiving 95% and 100% of the prescribed dose, it is also obvious that the differences between CT- and sCT-based plans are smaller than those between CT- and CBCT-based plans. Especially for V95%, there are almost no differences between CT- and sCT-based plans. For bladder and femoral heads, although the statistical differences between CT vs sCT and CT vs CBCT for dose are not as large as that for PTV, we can see that the dosimetric differences of sCT-based plans are smaller than those for CBCT-based plans, when compared to CT-based plans.

## Discussion

This work used a CycleGAN method to convert MV CBCT to sCT images for rectal cancer patients. The CT images at the treatment position were used as ground truth to evaluate the image quality, segmentation capacity and dose calculation capacity of sCT generated from CBCT acquired at the same position. **Figure 4** and **Table 1** revealed substantial image quality improvements. In the sCT images, the scattering artifacts were greatly diminished, and the organ boundaries were much clear than in the original CBCT images. This will not only helpful in the IGRT process for visual quality but also make contouring and dose calculation possible. The MAE was reduced from 135.84 ± 41.59 HU for the CT and CBCT comparison to 52.99 ± 12.09 HU for the CT and sCT comparison, which is a great improvement. The results are comparable to Harms et al.’s study, Liu et al.’s study and Lei et al.’s study for pelvic and abdominal regions (18, 20, 26). The MAEs for pelvic and abdominal regions were larger than that for brain of approximately 25 HU in Harms et al’ study and Lei’s study (20, 26). This is mainly because pelvic and abdominal regions are easily affected by respiratory motion, organ movement and organ filling status. In our study, although the CT and CBCT were acquired sequentially within a short time interval with the patient at the same position on the treatment couch. When the couch moves from the CBCT position to the CT position, the patient may move a small amount. In addition, respiratory motion may result in organ shape and position differences and differences in the patient’s outer boundary. The outer boundary difference can be seen in **Figure 4**. The SSIM index for CT and sCT comparison was 0.81 ± 0.03, which is much larger than the result of 0.71 ± 0.03 in Liu et al.’s study for abdominal images. The results indicate that our model greatly preserved the anatomy when improving the image intensity. Similar results can be found in Liang et al.’s study with an SSIM index of 0.85 for head and neck patients’ images where the structures are stable (21).

We are aiming to use sCT for online adaptive radiotherapy, in which the auto-segmentation and dose calculation capabilities are both important. In this study, we applied the autosegmentation model trained by using CT images to the sCT images and used the DSC index to evaluate the model performance on sCT images. From the DSC index between auto and manual-modified contours shown in **Table 2**, we can see that the autosegmentation model performance for femoral heads was very accurate. Although small modifications need to be made to the autosegmentation of the CTV and bladder, it is accurate enough comparing to most of the model performance. It is very helpful for improving contouring efficiency in ART process. For the CTV, the modifications were mostly made at the anterior boundary and boundary at foot direction. The scatter artifact was much larger in the middle part of the body for MV CBCT, which can affect the image quality of sCT generated based on MV CBCT and the autosegmentation performance of models trained on CT images. From the DSC index between autocontours on sCT and CT shown in **Table 2**, we can also see that the aotucontours between sCT and CT have some differences. May be directly use sCT images as a training dataset can obtain a better contouring model which worthy of further investigation.

To evaluate whether the sCT is capable of accurate dose calculation, sCT-based plans were compared to corresponding CT-based plans, and for comparison, CBCT-based plans were also compared to CT-based plans. The results show that no large dosimetric differences were found between sCT- and CT-based plans, while large differences were found between CBCT- and CT-based plans, especially for PTV. The dosimetric differences between sCT- and CT-based plans may be caused by the body size difference and larger motion artifacts of CBCT due to the longer acquisition time. To minimize motion artifacts, a motion management method can be used during CBCT acquisition, such as the surface guide light system, which has no direct contact with the patient’s body and will not make the patient uncomfortable. This topic requires further investigation.

In this study, we used a CycleGAN method to generate sCT based on CBCT to make sCT capable of adaptive radiotherapy. The model was trained using rectal cancer patients’ images. So the model can only be used for rectal cancer patients, which is an limitation of the cycleGAN method. It strictly depends on the training dataset. In order to improve the generalization of the cycleGAN model, we should include more images of different tumor sites in the training data. In Maspero et al.’s study, they have realized image transformation from kV CBCT to CT for HN, breast and lung cancer patients using a single model (27). To train a universal model for MV CBCT to CT transformation is our future research direction.

The generated sCT images were evaluated from three key aspects: image quality, segmentation capability and dose calculation capability based on autoplanning technique of UIH TPS. All of the results show that the sCT images are comparable to CT images, ensuring that the use of sCT for ART is possible. Using MV CBCT-based sCT for ART has several advantages. First, it represents the actual patient position and anatomy at the treatment couch. Second, no deformable registration is needed during the ART process, which ensures that no registration error is introduced. Third, accurate autosegmentation can improve the efficiency of the ART process. All of these advantages make the sCT-based ART theoretically superior to the plan selection method and the method based on deformable registration. Although for the CT-linac in our department, we can directly use online CT for adaptive radiotherapy. For other linacs with only MV CBCT, the method introduced in this study can make ART a reality.

## Conclusion

In this work, a CycleGAN method was used to improve MV CBCT image quality to make it eligible for ART. This method relies on unpaired CT and CBCT images, making it easier to apply them in clinical situations. The image quality, auto-segmentation capability and dose calculation capability were evaluated. The results show that the sCT images were comparable to CT images. The generated high-quality sCT images can make IGRT easier and more accurate. The accurate dose calculation capability of sCT can make DGRT and ART possible based on the actual patient anatomy at the treatment position. The proposed methods solved a key problem for rectal cancer ART realization based on MV CBCT.

## Data Availability Statement

The raw data supporting the conclusions of this article will be made available by the authors, without undue reservation.

## Ethics Statement

The studies involving human participants were reviewed and approved by Medical Ethics Committee of Fudan University Shanghai Cancer Center. The patients/participants provided their written informed consent to participate in this study.

## Author Contributions

JZ drafted the manuscript, did the image evaluation and provided suggestions on model training. ZC did the CycleGAN model training. JW provided suggestions on model training. FX did the manual contour modification. JP did the plan optimization. YH did the patient treatment and image acquisition. WH and ZZ designed the study and provided directive opinions. All authors contributed to the article and approved the submitted version.

## Funding

This work was supported by research funding from the National Natural Science Foundation of China (11905035).

## Conflict of Interest

The authors declare that the research was conducted in the absence of any commercial or financial relationships that could be construed as a potential conflict of interest.

The reviewer SD declared a shared affiliation with the authors to the handling editor at the time of review.
